# Serological Survey and Initial Control of *Mycoplasma gallisepticum* and *Mycoplasma synoviae* Infections in Breeder Flocks of Chinese Native Chickens

**DOI:** 10.3390/pathogens15070751

**Published:** 2026-07-17

**Authors:** Yan Yu, Mei Liu, Ying Zhang, Xinyue Shen, Jianmei Li, Qin Yang, Mei Xue, Yabin Dai

**Affiliations:** Jiangsu Institute of Poultry Science, Yangzhou 225125, China

**Keywords:** *Mycoplasma gallisepticum*, *Mycoplasma synoviae*, Chinese native chicken, seroprevalence, biosecurity measure, initial control

## Abstract

*Mycoplasma gallisepticum* (MG) and *Mycoplasma synoviae* (MS) are regarded as the most clinically and economically important avian *Mycoplasma* species, posing significant challenges to the poultry industry worldwide. In this study, MG and MS antibodies were detected by ELISA in 857 unvaccinated Chinese native chickens from 21 breeder flocks on four multi-age farms. The overall seropositive rates were 86.3% for MG and 98.4% for MS. Offspring from an infected breeder flock were reared under different environmental conditions, and serological monitoring was conducted over a 68-week production cycle. The antibody titers and seropositive rates of both mycoplasmas in birds reared in an isolation room were significantly lower than those in birds reared on the farm after maternal antibodies vanished. By adopting strict biosecurity measures, MG and MS infections were effectively controlled in an offspring flock derived from infected breeders, as evidenced by a substantial decrease in seropositive rates. The results suggested that MG and MS infections were prevalent in the breeder flocks of Chinese native chickens. Horizontal rather than vertical infection was the primary cause of high flock infection levels under field conditions. Strict biosecurity measures could be adopted to initially control MG and MS infections in offspring from the breeder flocks with high infection levels.

## 1. Introduction

Avian mycoplasmosis is a collection of diseases of worldwide distribution caused by bacteria in the genus *Mycoplasma* affecting several bird species. Mycoplasmas are members of the family Mycoplasmataceae, order Mycoplasmatales, and class Mollicutes. More than 20 *Mycoplasma* species have been recognized in avian hosts; however, only 4 species are considered pathogenic in poultry [1]. *Mycoplasma gallisepticum* (MG) and *M*. *synoviae* (MS) are pathogenic for chicken, turkey, and other species, and *M. meleagridis* and *M. iowae* are pathogenic primarily in turkeys. Currently, MG and MS are considered the most important pathogenic *Mycoplasma* species in the chicken farming industry from clinical and economic perspectives [1,2,3]. They are also listed as important pathogens for poultry by the World Organization for Animal Health (WOAH) [4].

Both MG and MS are transmitted vertically and horizontally, and lead to lifelong infections [1,3,5]. MG is the most pathogenic and economically significant mycoplasmal pathogen of chickens [6]. MG infection is known as chronic respiratory disease (CRD) or “air sac disease”, which describes a severe airsacculitis of chickens. Economic losses from reduced egg production and feed efficiency, drops in hatchability, and increased condemnations or significant downgrading of carcasses at slaughter make MG one of the costliest infectious diseases confronting today’s commercial chicken production worldwide [5,7,8]. MS infection most frequently occurs as a subclinical upper respiratory infection of chickens. It may cause acute to chronic respiratory disease, infectious synovitis, egg production losses, and eggshell abnormalities [1,9,10,11]. Respiratory disease resulting from MG or MS infection may be exacerbated by concomitant infection with a respiratory virus (e.g., infectious bronchitis virus or Newcastle disease virus) or a bacterium (usually *Escherichia coli*) [1,12].

Many serological and etiological studies have evidenced that MG and MS infections are widely prevalent in chicken flocks in China [13,14,15,16,17,18,19]. The infections have caused higher mortality rates, increased vaccination and treatment costs, and greater susceptibility to secondary bacterial or viral infections, leading to significant economic losses in the poultry industry [13,15]; this should prompt the development of effective control and prevention strategies. In this study, the serological method was employed to investigate the prevalence of MG and MS infections in breeder flocks of Chinese native chickens and to assess the dynamics of MG and MS infections within flocks reared under different environmental conditions. Subsequently, the MG and MS infections were initially controlled in an offspring flock from the infected breeders. The results obtained in the present study may help formulate strategies for the prevention and control of the diseases.

## 2. Materials and Methods

### 2.1. Serological Survey

Serum samples were collected randomly to detect antibodies against MG and MS in hens from 21 breeder flocks of Chinese native chickens on four multi-age farms (Farm A, B, C, and D) in Jiangsu and Hubei provinces, China. The distances between breeder flock locations on the farms varied from 20 to 30 m. All flocks were over 4 months old. They had no previous history of avian mycoplasmosis vaccination, but had been immunized against other diseases according to the conventional immunization program. During the early stages of development, birds were administered antibiotics for a short period to prevent bacterial infections; the antibiotic types varied by farm. For each flock, birds were selected from randomly selected cages, one bird per cage, and bled from the wing vein. All birds showed no obvious clinical symptoms at sampling. Serum samples were separated by centrifugation at 3000 r/min for 10 min and stored at −80 °C until further use.

### 2.2. Monitoring of Antibody Dynamics over a Production Cycle

One thousand newly hatched chicks were hatched from eggs collected from the breeder flock of Green-shelled egg chicken at 68 weeks of age on Farm A, where sporadic cases of CRD and synovitis have occurred in the flocks over the years. This breeder flock showed higher seropositive rates of MG and MS in the serological survey. After being wing banded, the chicks were randomly divided into two groups, with 100 birds in the experimental group (Group E) and 900 in the control group (Group C). Group E was maintained in an environmentally controlled isolation room without high-efficiency particulate air filtration. The room was located within the Jiangsu Institute of Poultry Science, with no other chicken flocks nearby, and had been thoroughly cleaned, washed, and fumigated with formaldehyde in advance. Throughout the experiment period, the birds were not administered any medication or immunization. The room and facilities were regularly cleaned but not disinfected. According to the requirements of epidemic prevention and control, all efforts were made to prevent birds from exposure to external pathogens, including, but not limited to, ultraviolet disinfection of personnel and materials before their entry. Group C was housed in a closed, environmentally controlled building on the original farm. The house had been cleaned and disinfected before the birds were placed in it. During rearing, the birds were managed according to the original procedures. Overall, biosecurity measures were inadequate, as mainly manifested in lax implementation of disinfection systems and movement restrictions for personnel and materials. Birds were administered doxycycline (Ruipu (Tianjin) Bio-pharmacy Co., Ltd., Tianjing, China) by drinking water at a concentration of 0.03% for prophylaxis from hatching to 6 days of age, and immunized with commercial live attenuated or inactivated vaccines against other diseases, including Marek’s disease, Newcastle disease, infectious bronchitis, infectious bursal disease, egg drop syndrome, and H5, H7, and H9 subtypes of avian influenza, according to the conventional immunization program.

Birds in both groups were cage-reared and received commercial feed and water *ad libitum*. The feed was purchased from Jiangsu Tiancheng Technology Group Co., Ltd. (Nantong, China) and mainly composed of corn, wheat, soybean meal, methionine, lysine, calcium hydrogen phosphate, stone powder, and premix containing vitamins and trace elements. The density of birds gradually decreased with age, with two birds per cage during the egg-laying period, according to conventional methods. Beginning at hatching, 20 and 30 birds were randomly chosen from different cages in Groups E and C, respectively, and bled for serological detection at different intervals up to 68 weeks of age. Some birds, especially in Group E, were bled more than once due to the high sampling frequency. Blood samples were collected from the jugular vein at hatching and from the wing vein thereafter. Serum samples were separated by centrifugation at 3000 r/min for 10 min and stored at −80 °C until analysis.

### 2.3. Decaying Pattern of Maternally Derived Antibodies in Chicks

Fifty Green-shelled egg chicks were hatched from eggs collected from the infected breeders at 54 weeks of age in Group C mentioned above. They were identified by wing bands and reared for 6 weeks, maintaining all the hygienic measures in an environmentally controlled isolation room. Chicks were supplied with commercial feed and water *ad libitum*. They were not vaccinated or medicated during the experiment period. Beginning at hatching, the same 20 birds were selected and bled for serological detection at different intervals up to 42 days of age. The birds were bled from the jugular vein before 6 days of age and from the wing vein thereafter. Serum samples were separated by centrifugation at 3000 r/min for 10 min and stored at −80 °C until processing. At the end of the experiment, all serum samples from chicks were used simultaneously to detect MG and MS antibodies.

### 2.4. Initial Control

One thousand and one hundred Green-shelled egg chickens were hatched from eggs collected from the infected breeders at 68 weeks of age in Group C mentioned above. They were kept in another closed house equipped with an automatic environmental control system on Farm A. The house was far away from other infected flocks on the farm. Before new birds were placed, the depopulated premises had been left idle for more than a month after thorough cleaning and disinfection of the house and equipment. During the rearing period, the feeding, management, and immunization of the flock remained essentially the same as before. However, what was different from before was the implementation of stringent biosecurity measures for disease control purposes, primarily including regular cleaning and disinfection inside the house, as well as strict movement restrictions on personnel, equipment, and materials. At 20 and 40 weeks of age, 60 birds were randomly selected and bled from the wing vein for the detection of MG and MS antibodies. Serum samples were separated by centrifugation at 3000 r/min for 10 min and stored at −80 °C until processing.

### 2.5. Antibody Assay

Serum samples were determined for the presence of MG and MS antibodies by enzyme-linked immunosorbent assay (ELISA) using the commercial MG antibody test kit (IDEXX 99-06729) and MS antibody test kit (IDEXX 99-06728) (IDEXX Laboratories, Inc., Westbrook, ME, USA), respectively, according to the manufacturer’s instructions. Based on the absorbance values at 650 nm (A650), sample-to-positive (S/P) ratios were calculated. Serum samples with an S/P ratio greater than 0.5 (titer greater than 1076) were considered positive, and a ratio of 0.5 or lower (titer less than or equal to 1076) was considered negative, based on the manufacturer’s recommendations. For each sample, the S/P value and the respective antibody titer were calculated by the formula:

$$S/P\;=\;\frac{\mathrm{Sample}\;A650—\mathrm{negative}\;\mathrm{control}\;\mathrm{mean}\;A650}{\mathrm{Positive}\;\mathrm{control}\;\mathrm{mean}\;A650—\mathrm{negative}\;\mathrm{control}\;\mathrm{mean}\;A650},$$

Log_10_ Titer = 1.09 (log_10_ S/P) + 3.36,

Titer = Antilog of log_10_ Titer.

### 2.6. Statistical Analysis

Statistical calculations (S/P ratio, antibody titer and positive rate) and figures were performed using the computer program Microsoft Excel version 365. Differences in the antibody titers and positive rates between groups were evaluated by the independent samples *t*-test and Pearson’s chi-square test, respectively, with the help of IBM SPSS Statistics for Windows, version 22.0 (IBM Corp., Armonk, NY, USA). A *p* value < 0.05 was considered statistically significant.

## 3. Results

### 3.1. Seroprevalence of MG and MS in Unvaccinated Native Chicken Flocks on Different Breeding Farms

A total of 857 serum samples were collected from 21 breeder flocks of Chinese native chickens on four multi-age farms for the detection of MG and MS antibodies. The results demonstrated that MG and MS infections were present on all farms surveyed. The seropositive rates of MG in flocks were from 37.5% to 97.8%, from 58.3% to 100%, from 60.0% to 100%, and 100%, and those of MS were from 85.7% to 100%, 100%, 90.0% to 100%, and 100%, on four farms, respectively. The overall seropositive rate of MG was 86.3%, varying from 37.5% to 100%, while that of MS was generally high, around 98.4%, and ranged from 85.7% to 100% (Table 1). In some flocks, the high individual differences in antibody titers might be due to the birds being in different stages of infection at sampling.

### 3.2. Dynamics of Antibodies Against MG and MS in Chickens Reared Under Different Environments over the Whole Production Cycle

Longitudinal monitoring of antibodies against MG and MS was conducted in birds reared in the isolation room (Group E) or on the farm (Group C) across the whole production cycle to assess the dynamics of MG and MS infections within the flocks.

**Figure 1:**
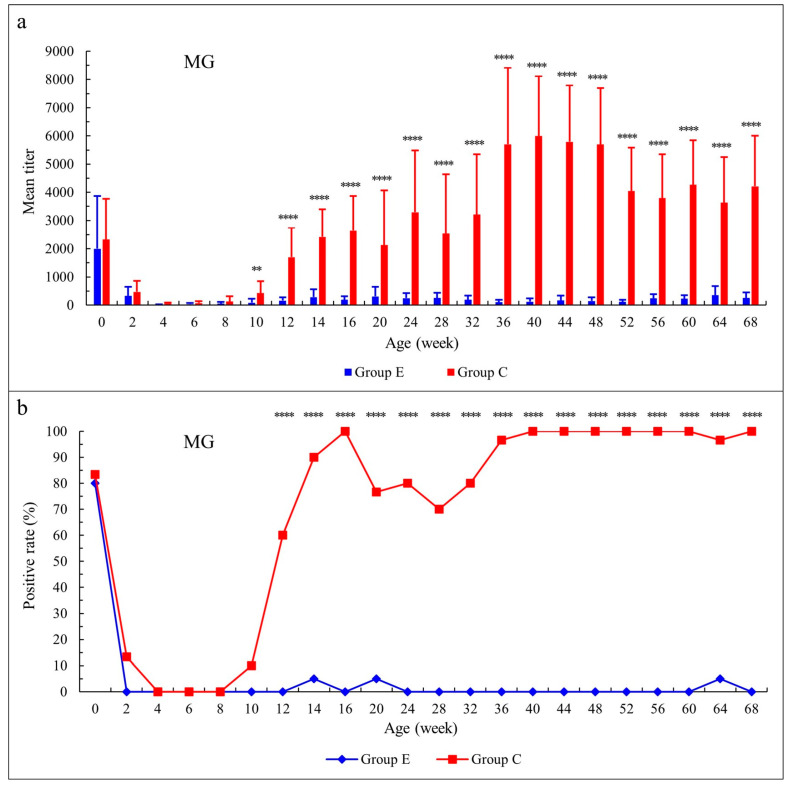
The dynamic changes in MG antibodies in chickens reared under different environmental conditions. (**a**) Antibody titer; (**b**) Seropositive rate. The birds were hatched from eggs collected from an infected breeder flock at 68 weeks of age. Group E was maintained in an isolation room, and Group C was in a house on the farm. At each time point, the antibody titer is expressed as mean ± SD. Statistical significance between the two groups is indicated by ** (*p* < 0.01) or **** (*p* < 0.0001).

For antibodies against MG, the mean titers in both groups decreased gradually after hatching, and the antibodies were virtually depleted at about 4 weeks of age. After 6 weeks of age, the mean titer in Group E increased slightly, but remained at a very low level until 68 weeks of age, while that in Group C gradually increased and was maintained at a high level until 68 weeks of age; the highest titer was observed at 40 weeks of age. There were no significant differences in mean titers between the two groups from birth to 8 weeks of age (*p* > 0.05), but from 10 weeks of age onwards, the mean titers in Group C were significantly higher than those in Group E (*p* < 0.01 or *p* < 0.0001) (Figure 1a). The seropositive rate in Group E declined from 80.0% at birth to 0 at 2 weeks of age; thereafter, it was 5.0% at 14, 20, and 64 weeks of age. The rate in Group C declined from 83.3% at birth to 0 at 4 weeks of age. It gradually increased after 8 weeks of age and reached 100% at 16 weeks of age. Following a slight decline from 20 to 36 weeks of age (with the lowest rate of 70.0% occurring at 28 weeks of age), the rate returned to 100% at 40 weeks of age and almost maintained this level until 68 weeks of age. No significant differences were observed in seropositive rates from birth to 10 weeks of age between the two groups (*p* > 0.05), while the rates in Group C were significantly higher than those in Group E after 10 weeks of age (*p* < 0.0001) (Figure 1b).

**Figure 2:**
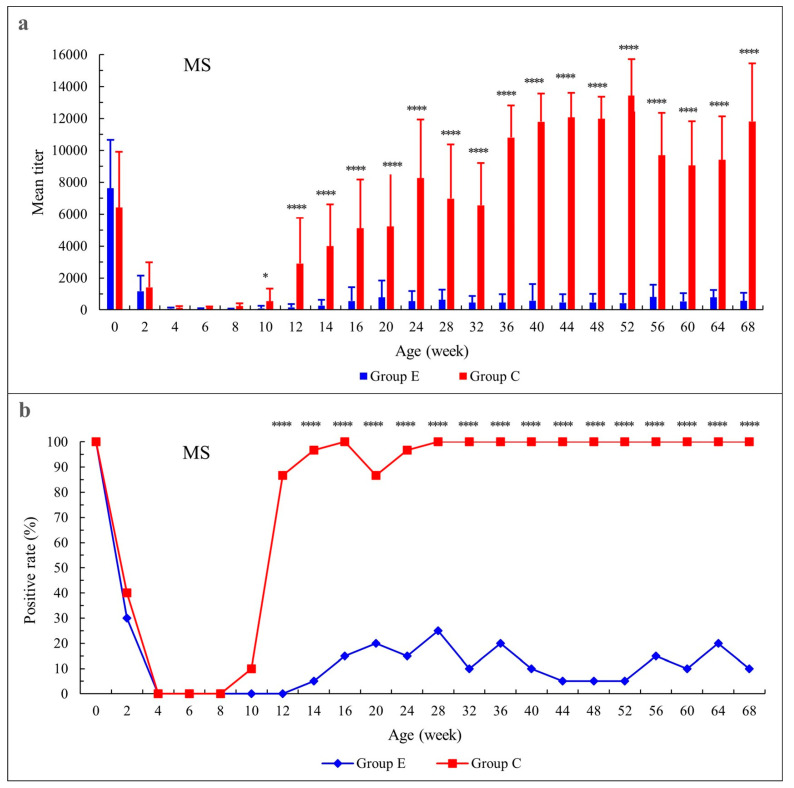
The dynamic changes in MS antibodies in chickens reared under different environmental conditions. (**a**) Antibody titer; (**b**) Seropositive rate. The birds were hatched from eggs collected from an infected breeder flock at 68 weeks of age. Group E was maintained in an isolation room, and Group C was in a house on the farm. At each time point, the antibody titer is expressed as mean ± SD. Statistical significance between the two groups is indicated by * (*p* < 0.05) or **** (*p* < 0.0001).

For antibodies against MS, the mean titers gradually decreased in both groups after hatching, and the antibodies were virtually depleted at about 4 weeks of age. After 8 weeks of age, the mean titer in Group E increased slightly, but basically remained at a very low level until 68 weeks of age. The mean titer in Group C gradually increased after 6 weeks of age and remained at a high level until 68 weeks of age; the highest titer was found at 52 weeks of age. There were no significant differences in mean titers between the two groups from birth to 8 weeks of age (*p* > 0.05), but the mean titers in Group C were significantly higher than those in Group E after 8 weeks of age (*p* < 0.05 or *p* < 0.0001) (Figure 2a). The seropositive rates declined from 100% at birth to 0 at 4 weeks of age in both groups. Thereafter, seropositive birds were detected in Group E after 12 weeks of age, and the seropositive rate varied from 5.0% to 25.0%; the highest rate was observed at 28 weeks of age. After 8 weeks of age, the seropositive rate in Group C gradually increased to 100% at 16 weeks of age, followed by a slight decline from 20 to 24 weeks of age, with the lowest rate of 86.7% at 20 weeks of age. Beginning at 28 weeks of age, all birds remained seropositive up to 68 weeks of age. No significant differences were observed in seropositive rates from birth to 10 weeks of age between the two groups (*p* > 0.05), while the rates in Group C were significantly higher than those in Group E after 10 weeks of age (*p* < 0.0001) (Figure 2b).

Overall, the dynamic changes in MG and MS antibodies were basically consistent within the same group. Since the chicks were hatched from an infected flock, it was apparent that the antibodies in birds about 4 weeks post-hatching were maternally derived. After maternal antibodies vanished, the antibody changes represented the immune responses of birds to pathogen infection. Either the antibody titers or the seropositive rates of MG and MS in Group C were significantly higher than those in Group E several weeks after maternal antibodies disappeared.

### 3.3. Decay of Maternally Derived MG and MS Antibodies in Chicks

**Figure 3:**
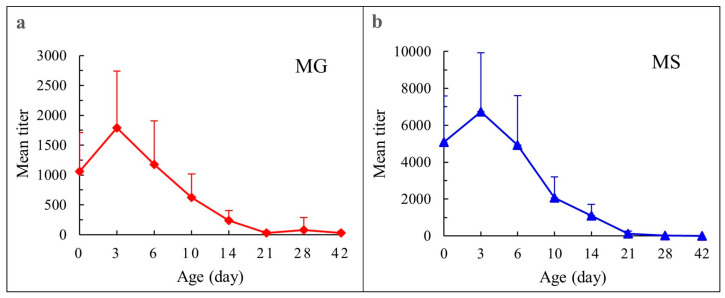
Decaying pattern of maternal antibodies against MG and MS. (**a**) MG; (**b**) MS. The chicks were hatched from eggs collected from an infected breeder flock at 54 weeks of age. For each time point, the antibody titer is expressed as mean ± SD.

Serum samples were collected successively at various intervals from hatching until 42 days of age to detect antibody levels against MG and MS in chicks. The decaying patterns of the maternally derived antibodies against the two pathogens were basically similar (Figure 3a,b). At 3 days after hatching, the antibody levels slightly rose; thereafter, they declined linearly as birds aged, and the antibodies were virtually depleted in birds at 21 days of age. Based on the change in titers between 3 and 14 days of age, the estimated individual half-lives of MG antibodies ranged from 3.1 to 4.4 days, with a mean of 3.8 days, and those of MS antibodies from 3.1 to 7.3 days, with a mean of 4.2 days.

### 3.4. Initial Control of MG and MS Infections in an Offspring Flock from the Infected Breeders

The MG and MS infections were attempted to be controlled in the progeny hatched from the infected flock by implementing strict biosecurity measures. At 20 weeks of age, no seropositive birds (0/60) were detected for MG, and the seropositive rate was 13.3% (8/60) for MS. At 40 weeks of age, the seropositive rates were 13.3% (8/60) for MG and 11.7% (7/60) for MS. The seropositive rates for both MG and MS substantially declined compared to those in their parent flock (Group C).

## 4. Discussion

As an economically significant poultry disease, avian mycoplasmosis causes immense losses to the poultry industry by decreasing egg production, reducing feed conversion efficiency, increasing mortality, downgrading carcass quality, and increasing vaccination and treatment costs [5,8]. Exacerbation of these production losses and mortality in birds may occur with concurrent secondary bacterial and/or viral infections [12,20]. The disease poses big challenges to the poultry industries worldwide.

The chickens surveyed in this study had not been immunized with avian mycoplasmosis vaccine, as this practice is not routine on these farms. Hence, the presence of antibodies to MG and MS was considered clear evidence that the birds had been naturally exposed to those two pathogens. In this study, the results of serological investigation showed that MG and MS infections were prevalent in the native chicken flocks, with seropositive rates of 86.3% and 98.4% for MG and MS, respectively. The results are approximately in agreement with those reported by Shen et al. [17] who observed the seropositive rates of MG and MS in breeder flocks were 95.7% (ranging from 77.5% to 100%) and 96.9% (ranging from 75.0% to 100%), respectively, in Anhui province, China, but slightly higher than those by Wang et al. [18] who found 54.8% and 82.3% of the seropositive rates for MG and MS, respectively, in breeder flocks in Shandong province, China. The seropositive rates of MG and MS may be associated with the age and breed of chickens, the epidemic situation of the diseases, hygiene and biosecurity measures for flocks, and the sensitivity of antibody detection methods. The rates usually increase with age [14,17,19]; this is confirmed by the dynamics of antibodies observed in the birds reared on the farm (Group C) in the present study.

It is well known that mycoplasmas can be transmitted both vertically (through infected fertile eggs) and horizontally (by direct and indirect contact, contaminated materials, personnel, and infectious aerosols or droplets). The vertical transmission rates are unpredictable and may vary between strains and phases of the infection [3,21]. The highest rates occur during the acute phase and are lower during the chronic phase of the infection [22]. The most recent studies showed that very low nucleic acid positive rates of both MG and MS were detected in dead or weak embryos and newborn birds from the infected breeders [17,18] with an exception reported by Yin et al. [23] who found only about 0.68% (2/294) of unhatched chicks were tested MS DNA positive, whereas 3.07% (10/325) of one-day-old weak chicks and 53.7% (158/294) of unhatched chicks MG DNA positive. In the present study, despite the lack of definitive etiological evidence, low seropositive rates for MG and MS in chickens reared in the isolation room (Group E) suggested that only a few birds might have been vertically infected. Even among the seropositive birds detected in the late stage, some were more likely to have been infected with pathogens shed by other infected birds in the group, or accidentally exposed to pathogens from other sources due to the long-term experimental period in a non-airtight isolation room, while others might be false positives due to the sensitivity and specificity of ELISA kits. The few vertically infected birds might be associated with birds hatched from eggs collected from breeders at 68 weeks of age, in the late stages of infection. In a study by Barbour et al. [24], the number of MG-infected embryos of hatching eggs collected from naturally infected broiler breeder flocks started to decline after 36 weeks of breeders’ age, reaching 6.7% infectivity at 57 weeks of breeders’ age. It is speculated that breeders at an older age may have higher protection against vertical transmission due to early natural exposure to field mycoplasmas, leading to higher immunity as birds age. Overall, under the conditions of this experiment, a few vertically infected birds did not lead to high prevalences of MG and MS infections in the non-high-density flock since only small numbers of mycoplasmas might have been shed by infected birds.

Unlike Group E, Group C was kept in a house on the original farm, and almost all birds had been infected with MG and MS. In addition to those from vertical transmission and shed from infected birds within the flock, birds in Group C might have been exposed to mycoplasmas that remained in the house before placement, as well as those that entered from the outside during rearing due to inadequate biosecurity measures. Furthermore, certain factors, such as other pathogen infections, may increase birds’ susceptibility to infections under field conditions. The combination of these factors might have resulted in high prevalences of MG and MS infections in Group C. Moreover, chickens of all ages are susceptible, but young ones are more prone to infection [1,25]. Birds are detected as seropositive by ELISA tests approximately 10 to 30 days after experimental infection with MG [26], but very low-dose infection during natural transmission may take much longer to yield a positive result. In this study, neither MG nor MS seropositive birds in Group C were detected until 10 weeks of age, after maternal antibodies had disappeared at 4 weeks of age, suggesting that the infections occurred a little late. Previous studies had shown that most MG or MS field isolates were susceptible to doxycycline [27,28]. These delayed detections of seropositive birds for MS or MG may be more likely associated with short-term use of the antibiotic doxycycline after hatching, but may also be due to the presence of maternal antibodies or to birds that had not yet been exposed to pathogens.

The vaccinated or infected breeder hens can transfer antibodies to their progeny through the egg yolk. Maternal antibodies are protective and can shield embryos against vertically transmitted pathogens during embryogenesis, as well as chicks for the critical first few weeks after hatching when their own immune systems are not yet fully mature [29,30,31]. As for mycoplasmosis, limited research data demonstrated that maternal antibodies could prevent or significantly reduce embryo mortality caused by virulent MG and MS [32,33], but conferred little protection against challenge of the virulent MG strain in chicks based on air-sac lesions post-challenge [21]; however, the correlation between antibody level and protective effect remains unelucidated. In the present study, maternal antibodies against MG and MS persisted for about 3 weeks in chicks. The half-life estimates of maternal antibody titers were 3.8 days and 4.2 days for MG and MS, respectively. This finding is approximately in agreement with the observations of Gharaibeh and Mahmoud [34], who reported that the half-lives of these antibodies were 4.9 days and 4.1 days for MG and MS, respectively, in broiler chicks.

Three general approaches can be adopted to control MG and MS infections: sourcing birds from MG- or MS-free breeder flocks and then preventing them from infection by good biosecurity during rearing, treating with antibiotics, and vaccinating with live attenuated or inactivated vaccines [5,35]. Though vaccination of broiler chicken breeders with a temperature-sensitive MG mutant vaccine (ts-11^®^) was reported to completely prevent infection by field MG and vertical transmission [24], both medication and vaccination may mitigate clinical signs and lesions, reduce mortality and production losses, and lower mycoplasma shedding and the risk of transmission, but do not necessarily eliminate infections from flocks [1,36,37,38,39,40,41,42,43]. Therefore, they may be useful measures for commercial flocks to control mycoplasma infections with the benefits of avoiding considerable economic losses, but are not ideal long-term solutions for breeder flocks. Additionally, continuous administration of an antibiotic may lead to the development of antibiotic resistance. The most effective control program is to establish MG- or MS-free breeder flocks. This program requires the flock to be managed and maintained under strict biosecurity to prevent the introduction of infections, and monitored regularly with serological testing to continually cull infected birds and confirm infection-free status.

Native chicken breeds are valuable genetic resources. In the present study, the native chicken breeds reared on the farms were introduced from their places of origin for germplasm conservation. In the current situation of high flock infection levels, the culling practice is infeasible because culling too many infected breeders is not conducive to breed conservation. Consequently, the elimination of MG or MS infection from flocks should be implemented in stages, with the first stage involving adequate measures to reduce the flock infection level. Biosecurity measures that are not disease-specific have been widely applied in preventing the transmission of diseases. In this study, stringent biosecurity measures against horizontal transmission were employed to control MG and MS infections in an offspring flock derived from the infected breeder flock based on the observation that horizontal rather than vertical infection can cause high flock infection levels in the progeny of infected flocks. These measures included thorough cleaning, washing and disinfection of premises and equipment, and leaving them vacant for more than one month to eliminate mycoplasmas from the previous infected flock before the new flock was placed, regular cleaning and disinfection of facilities to minimize the number of pathogens in the environment, and strict controlling the movement of personnel, equipment, and material to reduce the risks of disease introduction during the rearing period. Monitoring of MG and MS antibodies at 20 and 40 weeks showed that the seropositive rates of both MG and MS substantially decreased compared to those in their parents, suggesting the MG and MS infections had been preliminarily controlled. This provides favorable conditions for culling practices in the eradication of diseases.

## 5. Conclusions

In the present study, serological investigations showed that the positive rates of MG and MS antibodies in the breeder flocks were 86.3% and 98.4%, respectively, indicating that MG and MS infections were highly prevalent in Chinese native chickens. Long-term serological monitoring of MG and MS infections in birds reared in an isolation room and on the farm demonstrated that the rate of vertical infection of MG or MS was low, while horizontal infection was the main cause for high flock infection levels. Maternal antibodies against MG and MS persisted for approximately 3 weeks in chicks hatched from the infected breeders, with half-life estimates of 3.8 days and 4.2 days for MG and MS, respectively. The adoption of stringent biosecurity measures had effectively controlled MG and MS infections in an offspring flock derived from the infected breeder flock. A limitation of this study was the lack of etiological evidence for infection.

## Figures and Tables

**Table 1:** Seroprevalences of MG and MS in unvaccinated breeder flocks of Chinese native chickens.

Farm	Breed	Age (Day)	No. Sample	MG Antibody		MS Antibody	
				Titer *	Positive Rate (%)	Titer	Positive Rate (%)
A	Chongren	194	40	1235.2 ± 1253.74	37.5	6655.3 ± 3995.25	90.0
	Tibetan	350	42	3541.9 ± 2512.67	78.6	8446.6 ± 5243.63	85.7
	Gushi	257	40	2901.5 ± 1206.80	87.5	9674.3 ± 2507.19	100
	Green-shelled egg	237	46	5925.6 ± 2689.70	97.8	9845.4 ± 3865.38	100
B	Wenchang	150	40	2628.2 ± 1336.56	90.0	8064.3 ± 2199.79	100
	Qingyuan partridge	300	40	2732.5 ± 1693.39	80.0	9621.3 ± 3316.32	100
	Xianju	250	36	2312.3 ± 1633.58	83.3	5603.8 ± 1756.98	100
	Wenshang Luhua	220	36	3158.6 ± 2105.73	91.7	10,888.5 ± 3625.44	100
	White earlobes	320	48	2981.6 ± 3095.07	58.3	13,614.2 ± 3142.07	100
	Huainan partridge	130	44	2583.4 ± 1787.22	81.8	9837.9 ± 4361.81	100
	Gushi	190	42	2849.7 ± 1280.95	100	7204.3 ± 3502.47	100
	Xiaoshan	270	30	5840.1 ± 1182.61	100	13,335.4 ± 2521.62	100
C	Wenchang	187	40	2836.7 ± 1490.09	100	6241.9 ± 2367.30	100
	Longsheng phoenix	187	40	3276.1 ± 1756.05	90.0	4464.5 ± 1207.46	100
	Danzhou	210	40	1222.1 ± 1039.37	60.0	4012.0 ± 1997.91	90.0
	Miyi	210	40	3081.3 ± 2097.40	90.0	4560.2 ± 3072.21	100
	Yao	265	40	3508.4 ± 1382.20	100	6019.3 ± 3471.36	100
	Pudong	240	40	3176.6 ± 1582.17	90.0	5896.0 ± 2811.82	100
	Wuding	240	40	4050.9 ± 1467.60	100	5377.7 ± 3980.37	100
	Luyuan	240	43	5599.7 ± 1675.56	100	9403.0 ± 2936.72	100
D	Green-shelled egg	320	50	4961.9 ± 1891.67	100	10,658.6 ± 4059.53	100
Total	21		857		86.3		98.4

* The antibody titer is expressed as mean ± SD.

## Data Availability

All the data supporting the findings of this study are available from the corresponding author upon reasonable request.

## Abbreviations

The following abbreviations are used in this manuscript:

CRD	Chronic respiratory disease
ELISA	Enzyme-linked immunosorbent assay
MG	*Mycoplasma gallisepticum*
MS	*Mycoplasma synoviae*
WOAH	World Organization for Animal Health
